# The rise and fall of machine learning methods in biomedical research

**DOI:** 10.12688/f1000research.13016.2

**Published:** 2018-01-02

**Authors:** Hashem Koohy

**Affiliations:** 1 MRC Human Immunology Unit, Weatherall Institute of Molecular Medicine , University of Oxford, Oxford, UK; 2 Honorary Research Fellow in Computational Biology, Zeeman Institute, University of Warwick, Coventry, UK

**Keywords:** machine learning, linear regression, support vector machine, random forest, deep neural network, principal component, t-SNE, hierarchical clustering

## Abstract

In the era of explosion in biological data, machine learning techniques are becoming more popular in life sciences, including biology and medicine. This research note examines the rise and fall of the most commonly used machine learning techniques in life sciences over the past three decades.

## Introduction

Over the past three decades, biological data have grown dramatically in both size and complexity. The major contributors to the growth in size of computation biology data include, but not are not limited to, the ability of biologists to sequence complex genomes such as the human genome (1990–2003) ( Lander *et al.*, 2001), the advent of new high throughput sequencing techniques (around 2008) ( Marx, 2013), and most recently the very rapid advancements in single cell technologies, introduced in 2009 ( Wang & Navin, 2015).

The complexity of biological data has been growing even faster, and doesn’t seem to be linearly dependent on the size of data. Examples of complexity in the field of computational genomics include multiple diverse sources of technical noise, low signal to noise ratio, low numbers of biological replicates in comparative approaches, rare and usually hardly detectable mutations in non-coding regions and rare and barely identifiable cell types in complex heterogeneous systems such as the immune system and/or the brain.

At the intersection of mathematics, statistics and computer science is machine learning (ML), the *de facto* tool box in data science for deciphering the relationship between the input and output as well as detecting significant patterns within large, complex data sets. These quantitative approaches have been shown to be effective and are becoming increasingly popular in addressing challenges such as those outlined above. Highlights of their successful applications in functional genomics include, but are not limited to, learning and characterizing chromatin states by employing unsupervised approaches such as chromHMM ( Ernst & Kellis, 2012), predicting sequence specificities of DNA- and RNA-binding proteins using convolutional neural networks such as DeepBind ( Alipanahi *et al.*, 2015), and employing a combination of supervised and unsupervised approach to determine the genetic and epigenetic contributors of antibody repertoire diversity ( Bolland *et al.*, 2016). Nowadays it is almost impossible to publish a study on single cell assays without using dimensionality reduction methods such as Principal Component Analysis or t-SNE.

One indirect measure of the success of these techniques in extracting scientific insights from biological data is to measure the popularity and usage of machine learning algorithms in life sciences research over time ( Jensen & Bateman, 2011). Motivated by Jensen *et al.*, I therefore set out to update machine learning usage in life sciences. For this I quantified what fraction of published papers in the PubMed database mention a particular technique and how these number of citations are changed each year (see methods).

## Methods

For this analysis, I used the R RISmed package ( Kovalchik, 2015) to parse the publication data from NCBI. I examined publications in PubMed from 1990 to 2017 using a metric that measures the proportion of publications per year that mention the technique in the full text (Hits Per Year per Million articles published, or HPYM). The Popularity Rate (PR) of a technique was then defined as the difference between HPYMs in any two consecutive years. A positive PR shows an increase in popularity, whereas a negative PR reflects a decrease in popularity. I limited this note to 12 models listed in Table 1 which have been the most common or which showed a sharp change in popularity rate at a particular time. However, the R code is available with which any particular model during a specific period of time can be easily measured.

**Table 1.** Common Machine Learning Techniques in Life Sciences. This table shows 12 machine learning techniques whose popularity in life sciences have been investigated in this study. Technical note: Supervised means that the model requires training data to learn its parameters. A supervised model is used to predict the future instances. An unsupervised model doesn’t require any training data and is used to detect patterns within a dataset. Dimensionality reduction models are used to project high-dimensional datasets into lower dimension space where new variables are more interpretable.

Technique	Abbreviation	Category
Random Forest	RF	Supervised
Support Vector Machine	SVM	Supervised
Artificial Neural Network	ANN	Supervised
Deep Neural Network	DNN	Supervised & Unsupervised
Principal Component Analysis	PCA	Dimensionality Reduction
Linear Regression	LR	Supervised
Markov Model	MM	Unsupervised
Decision Tree	DT	Supervised
Hierarchical Clustering	HC	Unsupervised
t-Distributed Stochastic Neighbour Embedding	t-SNE	Dimensionality Reduction
Logistic Regression Model	LogReg	Supervised
Naïve Bayes Classifier	NBC	Supervised

## Results

**Figure 1.**
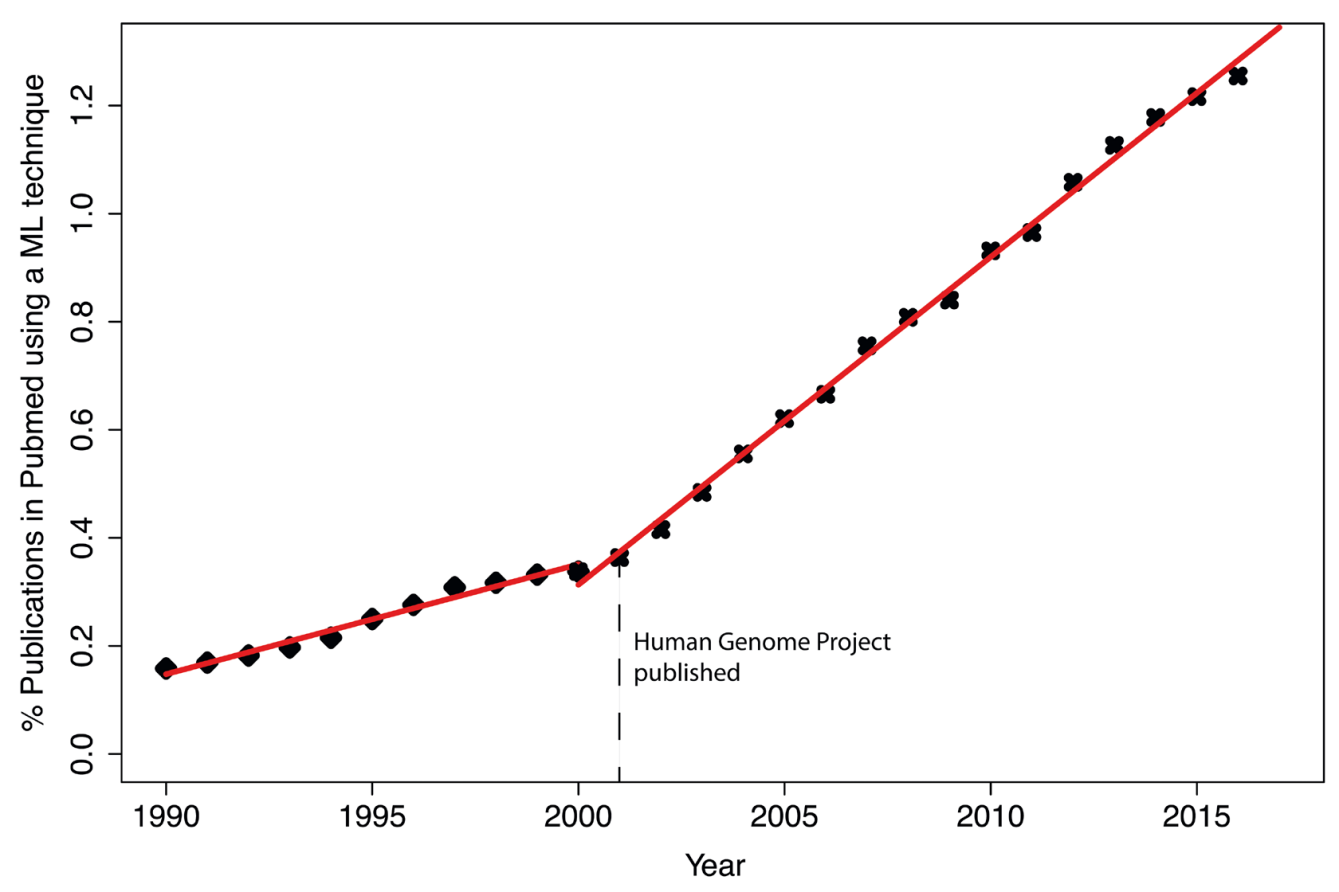
Cumulative usage of all 12 machine-learning techniques used in this manuscript. Two different linear regression models have been fitted to this data. The first one covers years from 1990 to 2000. The second one that shows a triple increase in its slope covers from 2001 till 2017. Y-axis shows the number of hits per 100 publications.

This analysis demonstrates that the overall popularity of machine learning methods in biomedical research has linearly increased since 1990 to 2017, but with two different slopes. From 1990 to 2000 the slope is 0.02, meaning that popularity increased only 2% per year. In 2001 (when sequencing big genomes became possible) the slope increased to 0.06, and since then it has remained constant. A maximum of 1.2% of all papers published in PubMed in any calendar year have mentioned one of the machine learning methods investigated in this study ( Figure 1). I was expecting to see a higher usage of ML in life sciences, but without a gold standard set to compare with, I would not be able to judge if this is too high or low or just about right.

**Figure 2.**
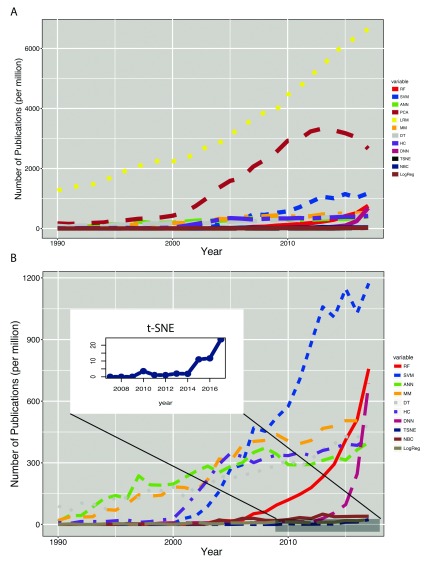
**A**: Trends of individual machine-learning techniques defined as per million hits in y-axis. **B**: Similar to **A** but without the two very highly used techniques Linear Regression and Principal Components Analysis in order to enhance clarity in usage of other not-very-commonly used techniques that were overshadowed by LRs and PCAs.

**Figure 3.**
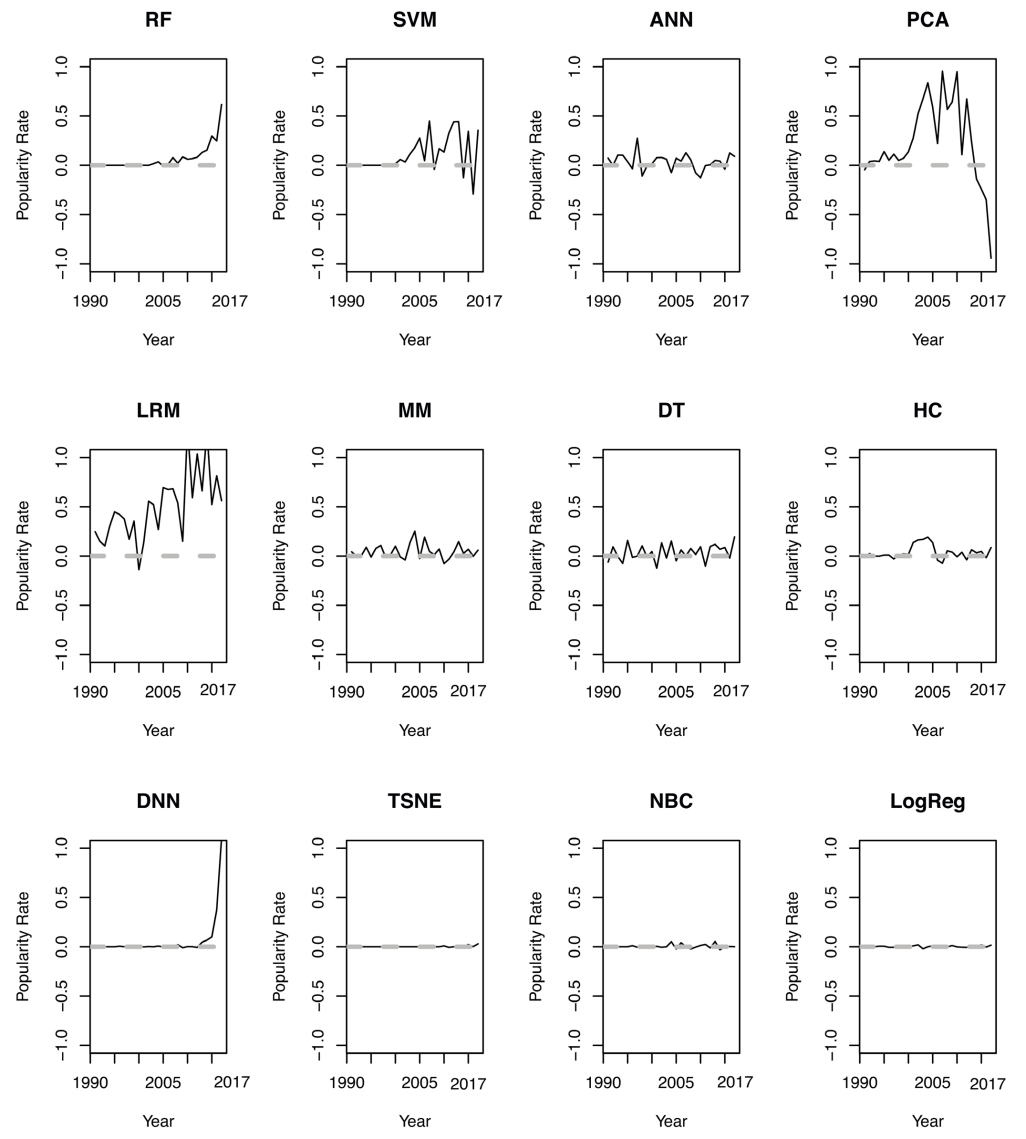
An illustration of popularity rate of all 12 techniques used in this manuscript. The PR has been defined as differences of HPYMs in each two-consecutive year for each model. This number have been further re-scaled to vary only between -1 and 1.

The Linear Regression (LR) models have been the most dominant machine learning techniques in the life sciences over the past three decades ( Figure 2A). It is interesting to see that LR models are still highly in used despite recent appearance of sophisticated ML techniques such as ensemble-based approaches and/or Support Vector Machines and even with very recent and state of the art deep learning techniques. Although, its popularity rate has been plateaued over the past few years ( Figure 3) meaning that its usage is increased linearly with a constant slope. With a constant increase of 300 HPYM, and considering its higher intercept at 1990, the linear regression models is predicted to be one of the most popular techniques over the next few years.

Perhaps a very surprising observation of this study is the rise and fall of Principle Component Analysis (PCA). PCA became very fashionable during 2000 to 2013. In fact, 3329 per million papers published in 2013 mentioned PCA which was the highest number of PCA usage. Since then it has been used less, although it still is the second most popular tool ( Figure 2A).

In early 2000s, unsupervised Hierarchical Clustering alongside newly introduced supervised techniques Support Vector Machines (SVMs) and Random Forests (RFs), showed a sharp rise in usage, which was mainly associated to microarray data analysis. Usage of hierarchical clustering plateaued shortly after its sharp popularity rise in 2000. SVMs kept their popularity longer, for almost a decade in fact, but subsequently dropped to an almost negligible popularity rate ( Figure 3). RFs on the other hand, showed less popularity at the beginning of their arrival, but later on (after 2013) they were ranked the second highest in popularity after Deep Neural Networks (DNN) ( Figure 2A, 2B and Figure 3).

During the period of 1990–2017, Artificial Neural Networks (ANNs) have demonstrated considerable fluctuations in popularity ( Figure 2B and Figure 3). ANNs in the early 1990’s after Linear Regression and PCA, were the most commonly used techniques until early 2000, when they lost their popularity to MMs, HCs and SVMs and even later to RFs. However, since 2013, a sub-family of ANN known as Deep Neural Networks (DNNs) made their way into the life sciences, and their usage since then has increased remarkably, so that DNNs currently have the highest popularity rate ( Figure 3).

The dimensionality reduction technique t-distributed Stochastic Neighbour Embedding (t-SNE) published in 2008, has become quickly tailored to all sorts of single cell techniques. It is therefore not surprising to see that t-SNE usage has also been very rapidly growing over the past few years ( Figure 2B). 

The text file contains the raw data underlying the results presented in this study, i.e. the number of publications in PubMed mentioning each machine learning technique from 1990–2017. These data is further normalized per million for downstream analysis Click here for additional data file. Copyright: © 2018 Koohy H 2018 Data associated with the article are available under the terms of the Creative Commons Zero "No rights reserved" data waiver (CC0 1.0 Public domain dedication).

## Discussion

I have illustrated the rise and fall of ML techniques in life sciences from 1990 to the present day. I chose this period because I believe this is the transition period for life scientists to join the big-data club. With the same R code used in this study to parse the publication data from NCBI, it would be possible to look at any period of time.

It was not very surprising to see LR models as the most commonly used model in the field, since:

a) LR models are one of the oldest ML methods that have been in use in almost any field,

b) Parameters in LR models can be learned by using a training data with just a few data samples.

c) A lot of other models can be placed under this umbrella, for instance by first applying a transformation function.

It was, however, surprising to see the sharp rise and fall of PCA. Perhaps a contributing factor to PCA being the most dominant dimensionality reduction method available in this period was its easy-to-use implementation in R. The question still remains as to why its popularity decreased from 2008 onwards. Perhaps the arrival of more versatile models such as RFs and SVMs which are very capable of handling high dimensionality and dealing with co-linearity in biological data eased the need to use PCA. Additionally, t-SNE as a tremendously growing dimensional reduction model in the field, is establishing itself as a strong competitor for the PCA.

ANNs have been fairly popular since the 1990s until around 2004. Around that time more readily useable and less complex techniques became available, such as SVMs, RFs and MMs. However, with the huge investments of giant information companies such as Google leading to very impressive applications of DNNs and other sub-families of ANNs, in various disciplines, DNNs, currently has the sharpest popularity rate ( Figure 3).

I appreciate that there are limitations to this study. For instance, for the majority of comparative analyses of gene expression, researchers use a differential expression software and/or package, but cite only the package name and not the underlying statistical or ML technique used in the package. These cases have not been covered in this study. However, this study can be considered as an approximation of the extent to which machine learning techniques are used in life sciences.

This note can be considered as an update of a similar study by Jensen *et al.* ( Jensen & Bateman, 2011), in which the authors investigated the rise and fall of a number supervised machine learning techniques in life sciences. Here, I have gone beyond the abstracts and searched the full text of each paper, for the usage of both supervised and unsupervised ML technique.

## Data and software availability

**Dataset 1:** The text file contains the raw data underlying the results presented in this study, i.e. the number of publications in PubMed mentioning each machine learning technique from 1990–2017. These data is further normalized per million for downstream analysis. DOI, 10.5256/f1000research.13016.d184022 ( Koohy, 2017).

R code used to parse the publication data from NCBI is available at: https://github.com/hkoohy/Machine_Learning_in_Life_Sciences

Archived source code as at the time of publication: http://doi.org/10.5281/zenodo.1039642 ( hkoohy, 2017).

License: GNU GENERAL PUBLIC LICENSE
