# Supplementary material for: The rise and fall of machine learning methods in biomedical research
Source: F1000Res. 2018 Jan 2;6:2012. Originally published 2017 Nov 10. [Version 2] doi: 10.12688/f1000research.13016.2 (PMC5760972; doi:10.12688/f1000research.13016.2)
Supplement: Peer review history

# Peer review: Referee response for version 2

**DOI:** 10.5256/f1000research.14767.r29390

**Published:** 2018-01-08

**Authors:** Konrad Förstner

**Affiliations:** 1 Core Unit Systems Medicine, Institute for Molecular Infection Biology, University of Würzburg, Würzburg, Germany

I thank Dr. Koohy for addressing my raised issues adequately.

I have read this submission. I believe that I have an appropriate level of expertise to confirm that it is of an acceptable scientific standard.

# Peer review: Referee response for version 1

**DOI:** 10.5256/f1000research.14114.r28432

**Published:** 2017-12-06

**Authors:** Konrad Förstner

**Affiliations:** 1 Core Unit Systems Medicine, Institute for Molecular Infection Biology, University of Würzburg, Würzburg, Germany

In the manuscript "The rise and fall of machine learning methods in biomedical research" the author has generated a quantitative perspective on the usage of machine learning methods in the life sciences. For some of the methods a hypothesis about the underlying reason for an increased or decrease popularity are discussed. The code for performing the analysis is available on GitHub and - like the retrieved PubMed data - has been deposited at Zenodo.

 I have several major objections / question / suggestion for the author:

- I tried to reproduce the analysis using RStudio 1.1.383 with the deposited RStudio project but got the following error when executing the R chunks in the file *Machine_Learning_Trends.Rmd*: "Error in library(informationRetrieval) : there is no package called ‘informationRetrieval’" The file *informationRetrieval.R* is located in another subfolder and I guess this just needs proper referencing inside of the project.
- The author states that he has selected widely used machine learning methods used in life sciences. I would have expected Naive Bayes classifiers in the list of most popular methods. A simple PubMed search for '"naive bayes classifier" OR "naive bayesian classifier' return twice as many hits as for "deep neural networks" (but over a longer time span):
  - https://www.ncbi.nlm.nih.gov/pubmed/?term=%22naive+bayes+classifier%22+OR+%22naive+bayesian+classifier%2
  - https://www.ncbi.nlm.nih.gov/pubmed?term=%22deep+neural+networks%22
- Similar issue for logistic regression: The analysis in the provided file *Machine_Learning_Trends.Rmd* actually contains the counting of publications containing logistic regression that shows a large (206,619 at the time of writing) and growing number of this but this method has not been discussed in the manuscript and is not displayed in the plots.
  - https://www.ncbi.nlm.nih.gov/pubmed?term=%22logistic%20regression%22
- The counting of hits for deep neural networks (DNN) is not done properly. Looking at the code to count the number of hits of different search terms shows that the author use "artificial neural networks" and "deep neural networks" and "deep learning" as search term for DNN (see code selection at the bottom of this section). I think using the search term "artificial neural network" for both ANN and DNN is not sound and changes the story of DNN (a special form ANN) significantly. Either DNN is treated as subset of ANN and only ANN are plotted or DNN and ANN are treated separately and the search term "artificial neural network" is not used for DNN. Furthermore the search term "deep learning" results in numerous unrelated hits before 2010 (e.g. PMID: 8936230, 9165817, 9487168, 10463930). https://www.ncbi.nlm.nih.gov/pubmed/?term=%22deep+learning%22 (then click on the "Result by year" histogram).
- The authors tries to explain the underlying reasons for the gain or loss of certain ML methods. In Figure 1 one of the publications of the human genome is placed in the year 2000 without any citation. The human draft genome was published in 2001 (International Human Genome Sequencing Consortium, Nature 409, 860–921, 2001, https://doi.org/10.1038/35057062) and it would be interesting to see what the author is referring to.
- The Popularity Rate (PR) introduced here is not plotted anywhere directly but is the slope of edges between the data points of two consecutive years. The author should consider visualizing this measurement of change as well.
- The curve plotted in Fig 1 A is nearly reassembled by the LRM curve in Fig 1 B. Is the observation in Fig 1 A maybe only an observation of the dominating LRM method? I do not understand why Fig 1 A can actually look nearly exactly like the LRM curve considering the other methods e.g. the PCA curve.

Code selection regarding ANN and DNN

 ```

 ANN_hits       <-  get_normliazed_number_of_hits(years = YEARs, query="artificial neural network[tw]", db="pubmed", normalization_value=1000000)

 NN_term <-  "(artificial neural networks[tw] OR deep neural networks[tw] or deep learning[tw])"

 DNN_hits <-  get_normliazed_number_of_hits(years=YEARs, query=NN_term, db="pubmed", normalization_value=1000000)

 ```

 Minor issues:

- Figure 1
  - Style: The different lines are hard to distinguish by color only - maybe consider an additional discriminator (e.g. dashed lines for a subset); Next to Fig 1 C is a lot of white space. Placing the t-SNE subplot to a different location (e.g. the middle of Fig 1 C) would make it possible to use this space more efficiently.
  - Maybe think rearranging the whole figure. Figure 1 C is a subplot of figure 1 B like the t-SNE plot is a subplot of Figure 1 C
- "de facto" should be written in in italic font
- The link to RISmed should use the link indicated at the page itself that says "Please use the canonical form https://CRAN.R-project.org/package=RISmed to link to this page."
- For Linear Regression Model sometimes "LRM" and sometime "LR" is used in the manuscript
- In order to understand which biological approaches / questions that are influencing the usage of different ML method the association of those methods with certain MeSH term would be interesting. Either as part of this manuscript or a future one.

I have read this submission. I believe that I have an appropriate level of expertise to confirm that it is of an acceptable scientific standard, however I have significant reservations, as outlined above.

# Response

**Published:** 2017-12-22

**Authors:** Hashem Koohy

**Affiliations:** Oxford University

I thank Dr Konrad Forstner for his time in evaluating the manuscript and for his very detailed comments/suggestions that I believe will immensely enhance the quality of the manuscript.

In the following I address the issues raised by Konrad.

In the manuscript "The rise and fall of machine learning methods in biomedical research" the author has generated a quantitative perspective on the usage of machine learning methods in the life sciences. For some of the methods a hypothesis about the underlying reason for an increased or decrease popularity are discussed. The code for performing the analysis is available on GitHub and - like the retrieved PubMed data - has been deposited at Zenodo.

I have several major objections / question / suggestion for the author:

- I tried to reproduce the analysis using RStudio 1.1.383 with the deposited RStudio project but got the following error when executing the R chunks in the file *Machine_Learning_Trends.Rmd*: "Error in library(informationRetrieval) : there is no package called ‘informationRetrieval’" The file *informationRetrieval.R* is located in another subfolder and I guess this just needs proper referencing inside of the project.

I have changed the package structure and added a cell on top of the rdm file so that it should easily find the *.r codes and source them.

- The author states that he has selected widely used machine learning methods used in life sciences. I would have expected Naive Bayes classifiers in the list of most popular methods. A simple PubMed search for '"naive bayes classifier" OR "naive bayesian classifier' return twice as many hits as for "deep neural networks" (but over a longer time span):
  - https://www.ncbi.nlm.nih.gov/pubmed/?term=%22naive+bayes+classifier%22+OR+%22naive+bayesian+classifier%2
  - https://www.ncbi.nlm.nih.gov/pubmed?term=%22deep+neural+networks%22

*In my very initial analysis I had naïve bayes classifier, but to my surprise after normalization  it was overshadowed by other highly used techniques. I therefore took it out.*

*Now, for the completion, I have added it again.*


* *

- Similar issue for logistic regression: The analysis in the provided file *Machine_Learning_Trends.Rmd* actually contains the counting of publications containing logistic regression that shows a large (206,619 at the time of writing) and growing number of this but this method has not been discussed in the manuscript and is not displayed in the plots.

Similarly, I had logistic regression models in my initial analysis. And for the same reason I took it out from the final submission and left to the reader to test if they wish. It has been added again.

- https://www.ncbi.nlm.nih.gov/pubmed?term=%22logistic%20regression%22

- The counting of hits for deep neural networks (DNN) is not done properly. Looking at the code to count the number of hits of different search terms shows that the author use "artificial neural networks" and "deep neural networks" and "deep learning" as search term for DNN (see code selection at the bottom of this section). I think using the search term "artificial neural network" for both ANN and DNN is not sound and changes the story of DNN (a special form ANN) significantly. Either DNN is treated as subset of ANN and only ANN are plotted or DNN and ANN are treated separately and the search term "artificial neural network" is not used for DNN. Furthermore the search term "deep learning" results in numerous unrelated hits before 2010 (e.g. PMID: 8936230, 9165817, 9487168, 10463930). https://www.ncbi.nlm.nih.gov/pubmed/?term=%22deep+learning%22 (then click on the "Result by year" histogram).

I really apologize for this. I have corrected in the code, and changed the manuscript accordingly.

- The authors tries to explain the underlying reasons for the gain or loss of certain ML methods. In Figure 1 one of the publications of the human genome is placed in the year 2000 without any citation. The human draft genome was published in 2001 (International Human Genome Sequencing Consortium, Nature 409, 860–921, 2001, https://doi.org/10.1038/35057062) and it would be interesting to see what the author is referring to.

I apologize again for the confusion. I was in fact referring 2001 IHGSC paper, as I had cited in the manuscript. I have changed the figure to make it clear.

- The Popularity Rate (PR) introduced here is not plotted anywhere directly but is the slope of edges between the data points of two consecutive years. The author should consider visualizing this measurement of change as well.

Very valid point. I have restructured the manuscript and the figures. Now, I have a separate figure for this.

- The curve plotted in Fig 1 A is nearly reassembled by the LRM curve in Fig 1 B. Is the observation in Fig 1 A maybe only an observation of the dominating LRM method? I do not understand why Fig 1 A can actually look nearly exactly like the LRM curve considering the other methods e.g. the PCA curve.
- Great observation. Both figures are very similar, though with different slopes and intercepts. In order to check if the cumulative figure is dominant by LRM, in a separate task, I filtered out LRM and made the cumulative figure. Although in both full-model and filtered-model we can see two different slopes (from 1990 to 2000, from 2001 ot 2017), not surprisingly, the full model fits better.

I think what happens is that around the time that  PCA starts declining, we have an almost exponential increase from other models such as RF, SVM and later on from DNN. These collectively delute effect of PCA decline.

Code selection regarding ANN and DNN

```

ANN_hits       <-  get_normliazed_number_of_hits(years = YEARs, query="artificial neural network[tw]", db="pubmed", normalization_value=1000000)

NN_term <-  "(artificial neural networks[tw] OR deep neural networks[tw] or deep learning[tw])"

DNN_hits <-  get_normliazed_number_of_hits(years=YEARs, query=NN_term, db="pubmed", normalization_value=1000000)

```

Minor issues:

- Figure 1
  - Style: The different lines are hard to distinguish by color only - maybe consider an additional discriminator (e.g. dashed lines for a subset); Next to Fig 1 C is a lot of white space. Placing the t-SNE subplot to a different location (e.g. the middle of Fig 1 C) would make it possible to use this space more efficiently.
  - Maybe think rearranging the whole figure. Figure 1 C is a subplot of figure 1 B like the t-SNE plot is a subplot of Figure 1 C
  - As suggested, I have restructure the manuscript and the figures. The manuscript now has three main figures which are hopefully more clearer than the previous version.
- "de facto" should be written in in italic font
- Corrected.
- The link to RISmed should use the link indicated at the page itself that says "Please use the canonical form https://CRAN.R-project.org/package=RISmed to link to this page."
- For Linear Regression Model sometimes "LRM" and sometime "LR" is used in the manuscript
- I have corrected for this.
- In order to understand which biological approaches / questions that are influencing the usage of different ML method the association of those methods with certain MeSH term would be interesting. Either as part of this manuscript or a future one.
- This is a very interesting point. Though as suggested is beyond this manuscript.

# Peer review: Referee response for version 1

**DOI:** 10.5256/f1000research.14114.r28048

**Published:** 2017-11-27

**Authors:** Alex Bateman

**Affiliations:** 1 European Molecular Biology Laboratory, European Bioinformatics Institute (EMBL-EBI), Cambridge, UK

I should firstly point out that I was co-author on the 2011 editorial published in Bioinformatics titled, “The rise and fall of supervised machine learning techniques” ^1^.  Therefore I was momentarily surprised to be invited to review a paper with such a similar title. That editorial was only a page and a half long and only really scratched the surface of the interesting topic of the prevalence of use of machine learning in the biosciences. The author cites our 2011 paper and mentions that the current article can be considered an update of it. However, that is only mentioned in the very final paragraph of the paper. It would seem reasonable to me to make that one of the first things mentioned in the paper.  Of course I am far from a neutral observer on this point.

 Overall I think that the article presents sound and interesting science and should be published within F1000Research. I think it provides a timely update to the 2011 editorial and expands it with some nice extra details. The article increases the number of ML methods investigated from 5 to 10.  Most notably linear regression models are included which top the league table.

 I noticed an inconsistency in the data presented for ANNs in this new paper compared to the 2011 paper. Why is that? The numbers for ANN are considerably lower in this article.  Is that because DNNs are split out from ANNs?  Throughout the paper it says that ANNs have become known as DNNs.  That is not correct. DNNs are a subtype of ANNs. So all DNNs are ANNs, but not all ANNs are DNNs.  That needs correction throughout.

 The following statement does not read well:

 "The sharp increase usage in popularity rate of DNNs over the past few years (Figure 1C) suggests that DNNs will take the PR lead again in the coming years."

 After multiple readings I would presume that PR lead means it has the highest popularity rate.  DNNs would have more than 300 more mentions per million papers per year.  Firstly that sentence is very confusing to understand for a reader.  For the first two readings I thought you were saying that DNNs would take the lead from LRMs, which would seem unlikely. On third reading I thought you meant that the slope of DNNs would overtake LRMs, but clearly it has already done that.  I think you should rethink that sentence or take it out.

**Minor points:**

 Page 2. At he intersection -> At the intersection

 Page 2. You mention that a surprising maximum of 1.2% of all paper mention one of the 10 ML techniques. Why is that surprising?  Is it too low, too high? Please explain.

 Page 2. NCBI database is mentioned.  NCBI has a lot of databases,  please specify which one.

 Page 3. less used less -> used less

I have read this submission. I believe that I have an appropriate level of expertise to confirm that it is of an acceptable scientific standard.

## References

[1] JensenLJBatemanA: The rise and fall of supervised machine learning techniques. *Bioinformatics*.2011;27(24) : 10.1093/bioinformatics/btr585 3331-2 10.1093/bioinformatics/btr585 22101152PMC3232371

# Response

**Published:** 2017-12-22

**Authors:** Hashem Koohy

**Affiliations:** Oxford University

I thank Dr. Alex Bateman for his time in evaluating the manuscript as well as for his very valuable comments.

In fact, I was inspired by Alex’s commentary. I therefore apologize for not appropriately mentioning this earlier in the manuscript. I have made this change to the manuscript and I hope that is clear enough now.

As Alex has suggested, I have made the distinction between ANNs and DNNs clear in corresponding paragraph and change the manuscript accordingly.

I have also addressed the minor points accordingly.

I hope the current version of manuscript meets Alex’s standards and consequently is clearer for the readers now.
